# Association between Single-Nucleotide Polymorphisms of the Tyrosine Kinase Receptor B (TrkB) and Post-Stroke Depression in China

**DOI:** 10.1371/journal.pone.0144301

**Published:** 2015-12-07

**Authors:** Zhiming Zhou, Xianhui Ding, Qian Yang, Jia Hu, Xianjin Shang, Xianjun Huang, Liang Ge, Taofeng Zhou

**Affiliations:** Department of Neurology, Yijishan Hospital of Wannan Medical College, Wuhu, Anhui Province, P.R. China; University of Naples Federico II, ITALY

## Abstract

**Background:**

Polymorphisms of the brain-derived neurotrophic factor (BDNF) have been investigated as candidate genes for post-stroke depression (PSD), and its receptor, neurotrophic tyrosine kinase receptor B (TrkB), has been associated with depression. However, no further data have yet reported the association between PSD and polymorphisms in TrkB. This study aims to investigate whether a relationship exists between TrkB polymorphisms and PSD.

**Methods:**

A total of 312 depression patients (PSD patients) and 472 non-depression patient controls (NPSD patients) were recruited. All patients were evaluated using the Hamilton Rating Scale for Depression (HAMD) to determine depression severity, and PSD patients were diagnosed in accordance with DSM-V criteria. Three single-nucleotide polymorphisms (SNPs), namely, rs1187323, rs1212171, and rs1778929, in the TrkB gene were genotyped by high-resolution melt analysis.

**Results:**

The SNP rs1778929 was significantly more associated with incident PSD in participants with the TT genotype than in those with CC (OR 0.482, 95% CI: 0.313–0.744). In terms of rs1187323, stroke was significantly more associated with incident depression in participants with the AC genotype than in those with AA (OR 0.500, 95% CI: 0.368–0.680). The minor allele (T) of rs1778929 (P = 0.024, OR = 0.725, 95% CI = 0.590–0.890) and the minor allele (C) of rs1187323 (P = 0.000, OR = 0.598, 95% CI = 0.466–0.767) were found to be significantly associated with PSD. Neither genotype nor allele frequencies of rs1212171 showed statistically significant differences between PSD and NPSD patients.

**Conclusions:**

The results suggest that rs1778929 and rs1187323 in the TrkB gene are significantly associated with post-stroke depression in the Chinese population. Further studies are necessary to confirm our findings.

## Introduction

Stroke is one of the most devastating amongst the neurological diseases. The disease is one of the top causes of mortality and disability-adjusted life-years lost globally, often causing death or gross physical impairment or disability [1]. Stroke has become the primary cause of death, particularly in developing countries [2]. Stroke survivors are often left with different degrees of disability, including physical and mental disabilities, such as post-stroke depression (PSD). The clinical symptoms of PSD mainly include apathy, fatigue, feelings of worthlessness, sleep changes, and anhedonia. PSD seriously affects the treatment and rehabilitation process and extends the period of hospitalisation [3].

At present, the pathogenesis of PSD is unclear. Previous studies show that PSD may be related to different factors, including behavioural, neurobiological, and social factors [4]. Amongst these factors, neurobiological changes after stroke may be specific for PSD. Neurotrophins are an important class of signalling molecules in the brain that are responsible for neuron growth, axon targeting, and maturation of synapses during development. Brain derived neurotrophic factor (BDNF) has been shown to perform an important function in the pathophysiology of depression. Previous studies have found that Low serum BDNF may indicate the development of PSD in patients with acute ischemic stroke [5], with several works showing decreased levels in depressed patients and recovery after antidepressant treatment [6, 7]. Recent evidence demonstrates a strong relationship between serum BDNF levels at admission and the development of PSD within 3 months [8].

BDNF fosters neuronal plasticity mainly by binding to a receptor complex formed by tyrosine kinase receptor (TrkB) [9, 10], the tyrosine kinase receptor (coded by NTRK2). A number of studies show that decreases in *N*-acetylserotonin contribute to the increased incidence of depression; *N*-acetylserotonin is a potent agonist to TrkB receptors, and the antidepressant and cognition-enhancing effects of *N*-acetylserotonin may be mediated by the activation of TrkB receptors [11]. This effect is due to *N*-acetylserotonin activating the TrkB signalling pathway in a circadian fashion (higher at night and lower during the day) via the TrkB receptor [11, 12]. The TrkB gene has been shown to perform a pivotal function in certain depression cases [13–15]. However, no previous studies have yet examined the relationship between TrkB polymorphisms and PSD. We hypothesize that some SNPs in the TrkB gene may be associated with PSD susceptibility. This study was conducted to determine the relationship between TrkB polymorphisms and PSD in stroke survivors.

## Materials and Methods

### Study Sample

This research was conducted in Yijishan Hospital of Wannan Medical College. Stroke patients were recruited from July 2012 to April 2014. A total of 986 patients with first-ever acute ischemic stroke were consecutively recruited within 7 days of stroke incidence. The inclusion criteria are as follows: (a) ischemic stroke confirmed by computed tomography (CT) scanning and/or magnetic resonance imaging (MRI); (b) the ability to complete the necessary investigations and questionnaires; (c) aged 18 years to 80 years; (d) 6-month follow-up completed and with available blood samples and clinical data; (e) the objective of the study was understood and informed consent was provided. Patients with a history of stroke and depression caused by other organic factors or history of substance abuse, bipolar disorder, psychotic disorders (such as schizophrenia or delusional disorders), severe aphasia or dysarthria, psychiatric illness, subarachnoid or intracranial haemorrhage, decreased level of consciousness, severe infections, inflammatory diseases, or dementia were excluded from this study.

The study protocol was approved by the Medical Ethics Committee of Yijishan Hospital of Wannan Medical College. Written informed consent was obtained from all patients.

### Diagnoses of PSD

Assessments were conducted by a single trained neurologist/ psychiatrist at approximately 2 weeks and at 1, 3, and 6 months after the onset of stroke to investigate the consequences of stroke at both the acute and chronic stages. The subjects were screened for depressive symptoms by using the 17-item Hamilton depression Rating Scale (HAMD) questionnaire. Subjects with a HAMD depression score of 8 or more were referred to trained researchers for further evaluation. Subjects with HAMD scores of ≥8 were further assessed with version 3.0 of the World Health Organisation Composite International Diagnostic Interview as the diagnostic criteria of depression after stroke. Patients with HAMD scores ≥8 and diagnosed with depression were divided into the depression group (PSD group), whereas those with HAMD scores <8 and HAMD scores ≥8 but not diagnosed with depression were classified into the non-depression group (NPSD group). PSD patients with diagnosed mental disorders were required to comply with the fifth edition of the US classification and diagnostic criteria for depression (DSM-V).

### Demographic and Clinical Characteristics

The following data were collected from each patient: age, gender, and risk factors for stroke, such as hypertension and diabetes, amongst others. All data were recorded according to the information obtained from the participants or their caregivers, and all participants underwent brain MRI or CT imaging as appropriate.

### Molecular Genetic Analysis

DNA was isolated from 200 μL blood samples by using columnar centrifugation with a whole blood genomic DNA extraction kit (Bioteke Biotechnology, Beijing, China) following the manufacturer’s protocol. Genotyping for the polymorphisms rs1187323, rs1212171, and rs1778929 was carried out.

### Genotyping

Three polymorphisms of the TrkB gene were genotyped by using high-resolution melt (HRM) analysis. PCR was performed and monitored via a CFX96 real-time PCR detection system (Bio-Rad), and melting data were analysed by Precision Melt Analysis software (Bio-Rad). Primers used included rs1187323 forward: 50-AGGCACTGCGGTGTATTTTC-30, rs1187323 reverse: 50- CATTTGCAAGCCTTGTCTGA-30; rs1212171 forward: 50- CCTAATTTTAAGTGGGAGATAGTGG-30, rs1212171 reverse: 50- AGAATAGGCAGTCTTACGGTGGT-30; and rs1778929 forward: 50- AACTTGGCTCCAAATCAACA-30, rs1778929 reverse: 50-CTGGGAGGGGTAGCATAAA-30. We amplified DNA fragments from 20 ng of genomic DNA. A full reaction system (20 μL) contained the following final reagent concentrations: 1 μL of genomic DNA (50 ng/μl), 1 μL of each primer, 10 μL of SsoFast EvaGreen Supermix (Bioteke Biotechnology, Beijing, China), and 7 μL of water. The amplification protocol consisted of an initial denaturation step at 95.0°C for 5 min, followed by 39 cycles of 95.0°C for 30 s, 95.0°C for 15 s, and 59.4°C for 30 s. Melting curves were generated by ramping from 65°C to 95°C at increments of 0.2°C for 10 s. Genotyping results were confirmed by sequencing random samples.

### Statistical Analysis

SNPs were analysed for associations with stroke by comparing minor allele frequencies as well as the constancy of the Hardy–Weinberg equilibrium in PSD and NPSD patients by using the chi-square test or Fisher’s exact test. The magnitude of association was expressed as OR with a 95% CI. *P* values <0.05 were considered statistically significant. All analyses were conducted using SPSS version 13.0 for Windows (SPSS Inc., Chicago, IL, USA). Haplotype frequency analyses were performed by SHEsis online software (http://analysis.bio-x.cn/myAnalysis.php). *P* values <0.05 were considered statistically significant.

## Results

**Fig 1:**
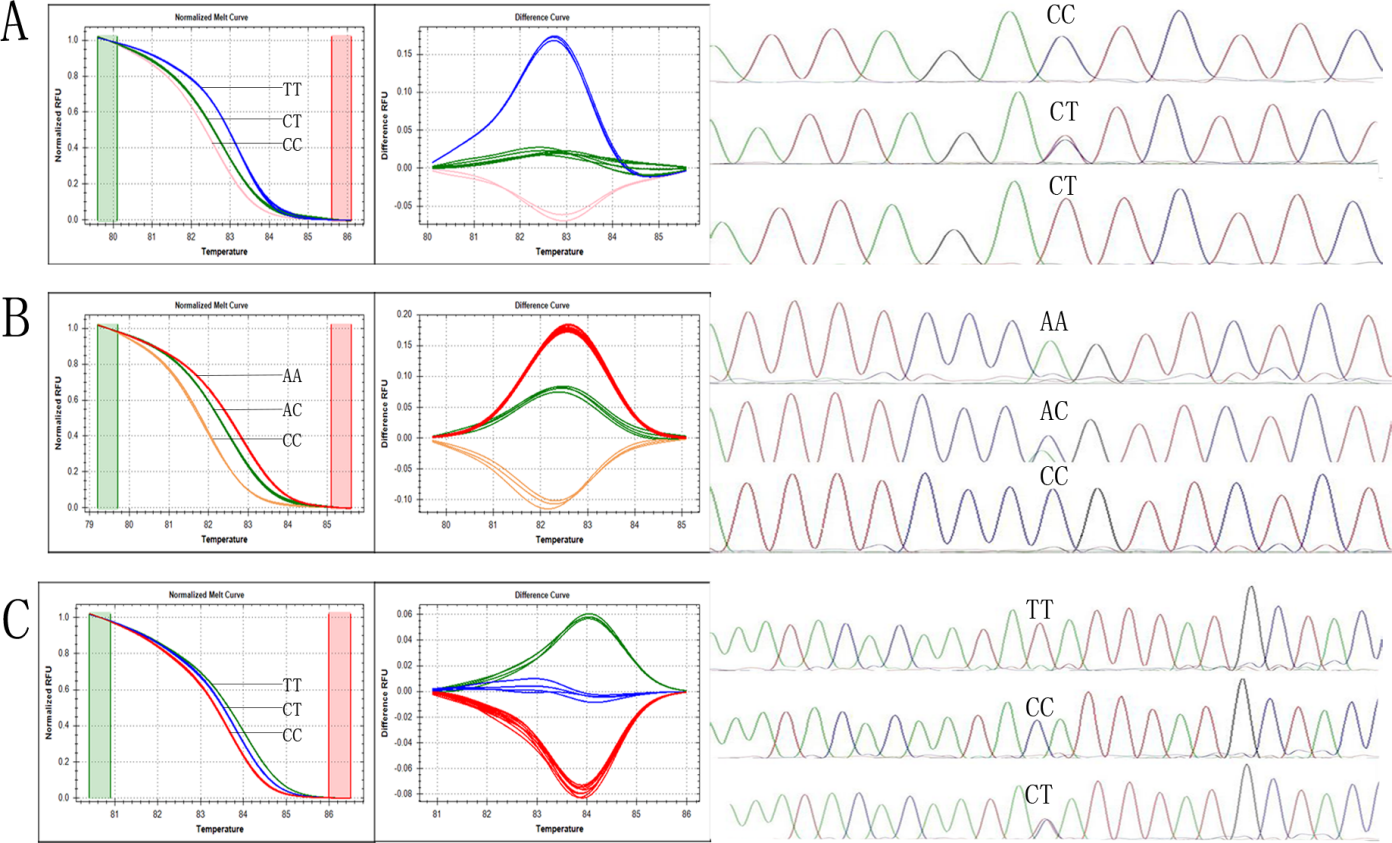
High resolution melting curves and sequencing pattern for different genotypes of three SNPs. A: rs1212171, B: rs1187323, C: rs1778929. SNPs were determined through HRM curves using the sequenced samples as control genotypes. The studied SNPs were successfully genotyped by HRM analysis, which shown in the left,and the right is sequencing pattern. The results obtained from the DNA sequencing analysis confirmed the reliability of the HRM assay.

In this study the genotype assignments of the three SNPs were determined through HRM curves using the sequenced samples as control genotypes. The studied SNPs were successfully genotyped by HRM analysis; the results obtained from the DNA sequencing analysis confirmed the reliability of the HRM assay. This was shown in Fig 1.

### The Incidence of PSD in 784 Patients

Nine hundred eighty-six ischemic stroke patients were enrolled in the present study. One hundred sixty-nine patients were lost to follow-up and Thirty-three died from ischemic stroke or other reasons. Seven hundred eighty-four patients (453 males and 331 females; mean age 64.1± 10.7 years) completed the 6-month follow up, including 312 depression patients (PSD patients) and 472 non-depression patient controls (NPSD patients), were recruited. The incidence of PSD in this sample was 39.8%.

### Distribution of TrKB Genotypes

In the present study, 312 PSD and 472 NPSD patients were recruited. The genotype and allelic frequencies of the three SNPs (rs1187323, rs1212171, and rs1778929) amongst PSD and NPSD patients were in Hardy–Weinberg equilibrium for all polymorphisms analysed (*P*>0.05). Neither genotype nor allele frequencies of rs1187323, rs1212171, and rs1778929 showed statistically significant differences between PSD and NPSD patients.

Tables 1 and 2 show the genotype and allele distributions of the three SNPs amongst PSD and NPSD patients. As indicated in Tables 1 and 2, neither genotype nor allele frequencies of rs1212171 showed statistically significant differences between the PSD patients and the controls. We found that rs1778929 is associated with PSD incidence (*P* = 0.004). The T allele was found in 45.5% of the chromosomes of the PSD patients in comparison with 37.7% of the chromosomes of the NPSD patients. The presence of the minor allele (T) increased PSD risk (*P* = 0.002, OR = 0.725, 95% CI 0.590–0.890). We also found that rs1187323 is associated with PSD incidence (*P* = 0.000). The C allele was observed in 25.2% of the chromosomes of the PSD patients in comparison with 16.7% of the chromosomes of the NPSD patients. The presence of the minor allele C increased PSD risk (*P* = 0.000, OR = 0.598, 95% CI 0.466–0.767).

**Table 1:** Genotype frequencies of TrkB gene in PSD and NPSD.

SNP	Genotype frequency, N (%)			χ^*2*^	P-value
rs1212171	CC	CT	TT		
PSD	53(16.99)	169(54.17)	90(28.85)	1.066	0.587
NPSD	94(19.92)	248(52.54)	130(27.54)		
rs1778929	CC	CT	TT		
PSD	93(29.81)	154(49.36)	65(20.83)	11.193	0.004
NPSD	175(37.08)	238(50.42)	59(12.50)		
rs1187323	AA	AC	CC		
PSD	169(54.17)	129(41.35)	14(4.49)	20.307	0.000
NPSD	330(69.92)	126(26.69)	16(3.39)		

TrkB, Tyrosine kinase receptor B; SNP, Single-nucleotide polymorphism; PSD, Post-stroke depression; NPSD, Non post-stroke depression.

**Table 2:** Allele frequencies of TrkB gene in PSD and NPSD.

SNP	Allele frequency, N (%)		χ^*2*^	P-value	OR(95%CI)
rs1212171	C	T			
PSD	275(44.07)	349(55.93)	0.679	0.410	0.918(0.749–1.125)
NPSD	436(46.19)	508(53.81)			
rs1778929	C	T			
PSD	340(54.49)	284(45.51)	9.464	0.002	0.725(0.590–0.890)
NPSD	588(62.29)	356(37.71)			
rs1187323	A	C			
PSD	467(74.84)	157(25.16)	16.602	0.000	0.598(0.466–0.767)
NPSD	786(83.47)	158(16.74)			

TrkB, Tyrosine kinase receptor B; PSD, Post-stroke depression; NPSD, Non post-stroke depression. SNP, Single-nucleotide polymorphism

Table 3 shows the association between stroke and incident depression stratified by TrkB genotype and strengthened progressively from the major homozygote through the heterozygote to the minor homozygote genotype. For rs1778929, stroke was significantly associated with incident depression in participants with the TT genotype (OR 0.482, 95% CI: 0.313–0.744). For rs1187323, stroke was significantly associated with incident depression in participants with the AC genotype (OR 0.500, 95% CI: 0.368–0.680).

**Table 3:** Association between SNP genotypes and the risk of PSD.

		Frequency N(%)				
SNP	Genotype (N)	PSD	NPSD	χ^2^	P-value	OR(95%CI)
rs1212171	CC(147)	53(36.05)	94(63.95)	1 ref		
	CT(417)	169(40.53)	248(59.47)	0.911	0.340	0.827(0.561–1.221)
	TT(220)	90(40.91)	130(59.09)	0.873	0.350	0.814(0.529–1.253)
rs1778929	CC(268)	93(34.70)	175(65.30)	1 ref		
	CT(392)	154(39.29)	238(60.71)	1.428	0.232	0.821(0.595–1.134)
	TT(124)	65(52.42)	59(47.58)	11.061	0.001	0.482(0.313–0.744)
rs1187323	AA(499)	169(33.87)	330(66.13)	1 ref		
	AC(255)	129(50.59)	126(49.41)	19.739	0.000	0.500(0.368–0.680)
	CC(30)	14(46.67)	16(53.33)	2.049	0.152	0.585(0.279–1.228)

SNP, Single-nucleotide polymorphism; PSD, Post-stroke depression; NPSD, Non post-stroke depression.

### Haplotype Analysis

We performed three SNP haplotype analyses (in the order of rs1212171, rs1778929, and rs1187323). Haplotype frequencies in the PSD group were compared with those in the NPSD population, and all frequencies below 0.03 were ignored during analysis. We found that the frequencies of the CCA, CCC, CTA, TCA, and TTA haplotypes are 0.187, 0.038, 0.064, 0.320, and 0.179, respectively, amongst the PSD patients, and 0.200, 0.070, 0.172, 0.291, and 0.169, respectively, amongst the NPSD patients. The TCA and TTA haplotypes were more frequently present in PSD patients than in NPSD patients and could be regarded as risk haplotypes (ORs: 1.145 and 1.068, respectively) (Table 4).

**Table 4:** Haplotype frequency analysis of rs1212171.rs1778929.rs1187323.

Allele			Frequency			
rs1212171	rs1778929	rs1187323	PSD (%)	NPSD (%)	*χ* ^2^	OR(95%CI)
C	C	A	116.59(0.187)	189.26(0.200)	0.446	0.916(0.709–1.184)
C	C	C	23.71(0.038)	66.21(0.070)	7.174	0.524(0.324–0.847)
C	T	A	39.64(0.064)	162.50(0.172)	39.462	0.326(0.227–0.469)
T	C	A	199.37(0.320)	274.57(0.291)	1.462	1.145(0.919–1.425)
T	T	A	111.40(0.179)	159.67(0.169)	0.231	1.068(0.818–1.393)

PSD, Post-stroke depression; NPSD, Non post-stroke depression.

The polymorphisms rs1778929 and rs1187323 were associated with PSD. Thus, we performed haplotype analyses of rs1778929 and rs118732. The frequencies of the CA, CC, TA, and TC haplotypes are 0.505, 0.039, 0.243, and 0.212 respectively, amongst the PSD patients and 0.492, 0.131, 0.341, and 0.036, respectively, amongst the NPSD patients. The TC haplotype was more frequently present in PSD patients than in NPSD patients and could be regarded as a risk haplotype (OR: 7.192) (Table 5).

**Table 5:** Haplotype frequency analysis of rs1778929. rs1187323.

Allele		Frequency			
rs1778929	rs1187323	PSD (%)	NPSD (%)	*χ* ^2^	Odds Ratio(95%CI)
C	A	315.42(0.505)	464.08(0.492)	0.289	1.057(0.863~1.294)
C	C	24.58(0.039)	123.92(0.131)	36.983	0.271(0.174~0.424)
T	A	151.58(0.243)	321.92(0.341)	17.149	0.620(0.494~0.778)
T	C	132.42(0.212)	34.08(0.036)	122.752	7.192(4.859~10.645)

PSD, Post-stroke depression; NPSD, Non Post-stroke depression

## Discussion

PSD is a frequent complication after stroke that can affect the quality of life of stroke patients and interfere with function recovery. This disease presents a high recurrence rate, high mutilation rate, and high incidence. In our study, the prevalence of PSD was found to be 39.8%, consistent with the results of previous research [16, 17]. A meta-analysis has estimated the pooled frequency of PSD to be 33% [18]. At present, despite the abundant literature available, the true prevalence rate of PSD remains difficult to define. Variability between studies arises not only from methodological problems during investigation but also from complexities associated with recognising, diagnosing, and assessing depression [19].

To the best of our knowledge, our research is the first study to report an association between TrkB polymorphisms and the risk of PSD in stroke survivors in a Chinese population. Results suggest that rs1778929 and rs1187323 are associated with PSD, that the minor allele increases PSD risk, and that the strength of association between incident stroke and depression increases incrementally with the number of minor alleles. PSD risk is higher in patients with the TT genotype of rs1778929 and the AC genotype of rs1187323 than in patients with other genotypes of these polymorphisms. These findings provide further evidence that the SNPs of TrkB play an important role in the aetiology of PSD.

Genome-wide association studies have identified several new and common genetic factors, including certain TrkB polymorphisms, which can lead to depression. For example, rs1212171 has been reported to be statistically significantly associated with the absence of depressive symptoms in HIV positive subjects [15]. Kohli’s study suggests that a combination of several independent risk alleles within the TrkB locus is associated with suicide attempts in depressed patients [20]. Previous research works have revealed a genotype-wise association between rs2289656 on TrkB and sporadic Alzheimer’s disease [21]. Another study showed that TrkB rs1187323 and TrkB rs1778929 display statistically significant differences in genotypic tests between geriatric depression and the corresponding control groups [14].

We performed haplotype analysis of rs1212171, rs1778929, and rs1187323 and found that TCA and TTA could be regarded as risk haplotypes for PSD. Because rs1778929 and rs1187323 were significantly associated with PSD but neither genotype nor allele frequencies of rs1212171 showed possible associations with PSD, we performed haplotype analyses of rs1778929 and rs118732. Haplotype analyses revealed that the haplotypes of rs1778929–rs1187323 present a highly significant and positive association with PSD. A major haplotype, TC, which is formed by rs1778929–rs1187323, was also discovered as a risk haplotype for PSD.

Although the mechanism of the effect of TrkB polymorphisms on the risk of PSD remains unknown, numerous investigators have shown that BDNF is associated with the pathogenesis of depression [22, 23], including PSD. BDNF, through its tyrosine kinase receptor, TrkB, is known to regulate neuronal plasticity and survival. However, the exact relationship between BDNF and PSD remains unclear, and little is known about SNPs, including rs1187323, rs1212171, and rs1778929, in the TrkB gene. Knowledge of these SNPs is essential to evaluate the neurotrophic properties of BDNF. The data presented in this study reveal correlations between the TrkB gene and PSD for the first time. Thus, we hypothesise that TrkB affects the occurrence of PSD by regulating the function of BDNF. The SNPs rs1778929 and rs1187323 may be potential candidates for protection against depression after stroke and require further examination in future work. The associations between TrkB polymorphisms and PSD observed in our study suggest the direct effect of these two SNPs on TrkB activity as well as corresponding effects on the pathogenesis of PSD. Further studies are necessary to explore whether rs1778929 and rs1187323 exert direct effects on TrkB expression or TrkB activity in the brain.

Our study presents several limitations. Some patients who presented more severe strokes, those who died before the 6-month follow-up, and those who developed PSD later than the prescribed time were excluded from this work and may limit the general applicability of our findings. Positive associations may have arisen through accidental stratification effects during sample collection. Thus, more studies with different samples must be conducted in future research. Finally, only three SNP variations are included in this study, but 2,076 known SNPs are located in the NTRK2 gene. More of these SNPs should be examined in future work.

In conclusion, our findings provide further evidence that the SNPs of TrkB performs an important function in the aetiology of PSD on the basis of single-locus analyses. Our findings support the hypothesis that SNPs from TrkB contribute to the risk of PSD in China. However, the underlying mechanism remains largely unknown. Therefore, independent replications with large sample sizes are necessary to confirm the roles of the TrkB genetic polymorphisms found in this study in PSD.

## Conclusions

Our results show that SNPs rs1778929 and rs1187323 in the TrkB gene are significantly associated with PSD in the Chinese population. However, the underlying mechanism remains unknown. Further studies are necessary to confirm our findings.

## Supporting Information

S1 Table Relevant data underlying the findings described in manuscript. (XLS)
